# Identification of a novel *THRB* mutation causing thyroid hormone resistance syndrome

**DOI:** 10.20945/2359-4292-2026-0006

**Published:** 2026-01-29

**Authors:** Jing Yang, Chuan Wang, Li Quan, Sheng Jiang

**Affiliations:** 1 State Key Laboratory of Pathogenesis, Prevention and Treatment of High Incidence Diseases in Central Asia, Urumqi, China; 2 Department of Endocrinology, The First Affiliated Hospital of Xinjiang Medical University, Urumqi, China; 3 Department of Endocrinology, The Xinhua Hospital of Yili Kazakh Autonomous Prefecture, China

**Keywords:** Thyroid hormone resistance, thyroid hormone receptor beta, mutation, thyroid hormone resistance syndrome, refetoff syndrome

## Abstract

Resistance to thyroid hormone syndrome (RTHS) is a rare disorder caused by mutations in the thyroid hormone receptor beta (*THRB*) gene, resulting in impaired action of thyroid hormones on target tissues and organs. We report a case of a 57-year-old Chinese male who presented with palpitations and hand tremors. Laboratory tests revealed elevated serum thyroid hormone levels, while serum thyroid-stimulating hormone (TSH) levels remained within the normal range. Enhanced magnetic resonance imaging of the pituitary gland showed no abnormalities. Through genetic testing, we identified a rare heterozygous point mutation in the *THRB* gene, specifically c.938T>C: p.M313T. To the best of our knowledge, this mutation site has not been previously reported in the literature. Clinically, RTHS is often misdiagnosed as hyperthyroidism, leading to inappropriate treatment and potential exacerbation of thyroid hormone resistance. Therefore, accurate diagnosis of this condition is crucial. Given the rarity of RTHS, we hope that this case report will enhance the understanding of its clinical manifestations and management, particularly in patients with *THRB* gene mutations.

## INTRODUCTION

Resistance to thyroid hormone syndrome (RTHS) is a relatively rare disorder, primarily caused by mutations in the thyroid hormone receptor beta (*THRB*) gene. These mutations lead to impaired responsiveness of tissues and organs to thyroid hormones ^(1,2^0. In the human body, thyroid hormones regulate various physiological processes through two main receptor genes: the *THRB* and the thyroid hormone receptor alpha (*THRA*) ^(3)^. There are three receptor subtypes, namely THRα1, THRβ1, and THRβ2, which are predominantly expressed in different tissues. The prevalence of RTHS is approximately 1 in 40,000 individuals ^(4,5)^. Clinical manifestations of RTHS are diverse and may include palpitations, hand tremors, and other symptoms.

The majority of RTHS cases are associated with mutations in the *THRB* gene and are termed RTHβ ^(6)^. A minority of cases are related to mutations in the *THRA* gene, as well as defects in genes involved in thyroid hormone transport and metabolism. Laboratory tests typically reveal persistently elevated levels of free T3 and free T4, while serum thyroid-stimulating hormone (TSH) levels remain within the normal range ^(7-9)^. The genetic inheritance pattern is predominantly autosomal dominant, although autosomal recessive inheritance and sporadic cases have also been reported. Notably, individuals with heterozygous *THRB* gene mutations often exhibit more severe symptoms compared with those with homozygous mutations.

Due to the varying degrees of thyroid hormone resistance in target tissues, patients with RTHβ exhibit high clinical phenotypic heterogeneity. They may present with symptoms of hyperthyroidism, hypothyroidism, or be entirely asymptomatic. However, most patients with RTHβ are asymptomatic, which can easily lead to missed or incorrect diagnoses in clinical practice, and subsequently exacerbate thyroid hormone resistance. Therefore, accurate diagnosis of this disease is of great significance.

In this study, through gene sequencing, we identified a rare mutation (p.M313T) in the *THRB* gene of a patient with RTHS. To the best of our knowledge, this is a novel gene mutation site, and there have been no previous reports of this mutation.

## CASE REPORT

A 57-year-old male presented to our outpatient clinic with a 3-year history of palpitations and hand tremors. Previous thyroid ultrasound examinations had indicated the presence of thyroid nodules; however, the results of his thyroid function tests were unavailable. During this period, the patient had not received any specific treatment. On physical examination, the patient’s height was 165 cm, weight was 83 kg, blood pressure was 113/72 mmHg, heart rate was 81 beats per minute, and respiratory rate was 18 breaths per minute. There was no exophthalmos or ophthalmic signs. The thyroid gland was not enlarged, no tremors were palpable, and no vascular murmurs were detected. The heart, lungs, and abdomen were normal, and there was no edema in the lower extremities. The patient had married at the age of 22 years and had two sons. His past medical history was unremarkable, and he had no other family members with similar problems.

**Figure 1:**
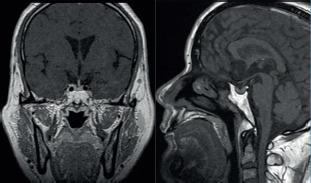
The proband underwent plain and enhanced magnetic resonance imaging of the pituitary gland. No abnormal signals were detected in the pituitary gland, and the pituitary stalk was centrally located. No abnormalities were observed in the morphology or signals of the optic chiasm and bilateral internal carotid arteries. On enhanced scanning, the enhancement of the pituitary tissue was relatively uniform and consistent, with no evidence of abnormal enhancement.

Thyroid ultrasound revealed multiple bilateral benign nodules (TI-RADS category 3). Laboratory tests performed on March 6, 2024, demonstrated elevated thyroid hormone levels with a normal TSH level (Table 1). A repeat thyroid function test conducted in our hospital on March 16, 2024, yielded similar results (Table 1). To further rule out the influence of assay reagents, an additional thyroid function test using chemiluminescence immunoassay was performed in a different hospital on March 21, 2024, which again showed elevated thyroid hormone levels with a normal TSH level (Table 1). The iodine uptake rate of the thyroid gland was 25.4% at 3 hours (reference range: 6&3x0025;–28%) and 58.1% at 24 hours (reference range: 16–50%). Magnetic resonance imaging (MRI) of the pituitary gland did not reveal any pituitary adenomas (Figure 1). Other pituitary axes showed normal function (Table 2). An electrocardiogram showed sinus rhythm. Based on the patient’s medical history and examination, RTHS was suspected, and the patient was advised to undergo genetic testing.

**Table 1:** Results of the proband’s thyroid function and antibody panel

Test (unit)	Result by Date		Normal range	Test (unit)	Result by Date	Normal range
	March 6, 2024	March 16, 2024			March 21, 2024	
TSH (mIU/L)	2.13	2.51	0.72–4.2	TSH (µIU/mL)	2.7082	0.3500–4.9400
FT4 (pmol/L)	57.1	52.4	12–22	FT4 (ng/dL)	2.13	0.70–1.48
TT4 (nmol/L)	193.00	171.00	66–181	TT4 (µg/dL)	15.53	4.87–11.72
FT3 (pmol/L)	14.4	13.00	3.1–6.8	FT3 (pg/mL)	6.41	1.58–3.91
TT3 (nmol/L)	3.71	3.31	1.2–3.1	TT3 (ng/mL)	1.78	0.64–1.52
TPOAb (IU/mL)	15.6	16.70	0–34	TPOAb (IU/mL)	2.42	<5.62
TgAb (IU/mL)	21.2	12.10	0–115	TGAb (IU/mL)	0.65	<4.11
TRAb (IU/L)	–	<0.80	0–1.75	TRAb (IU/L)	1.670	0.000–1.750
TG (ng/mL)	–	207.00	3.5–77	TG (ng/mL)	190.00	3.50–77.00
rT3 (U/L)	–	1.11	0.31–1.24	–	–	–

Abbreviations: FT3: free triiodothyronine; FT4: free thyroxine; rT3: reverse triiodothyronine; TG: thyroglobulin; TgAb: thyroglobulin antibody; TPOAb: thyroid peroxidase antibody; TRAb: thyrotropin receptor antibody; TSH: thyroid-stimulating hormone; TT3: total triiodothyronine; TT4: total thyroxine.

**Table 2:** Results of the proband’s laboratory tests

Parameter (unit)	Result	Normal range
Potassium (mmol/L)	3.58	3.5–5.1
Sodium (mmol/L)	139.56	137–147
Calcium (mmol/L)	2.10	2.1–2.55
Magnesium (mmol/L)	0.89	0.7–1.0
Phosphorus (mmol/L)	0.94	0.81–1.45
Creatinine (mmol/L)	68.6	53–115
Fasting glucose (mmol/L)	4.83	3.9–6.1
Triglycerides (mmol/L)	0.92	0.29–1.83
Total cholesterol (mmol/L)	4.97	2.8–5.7
LDL-C (mmol/L)	3.5	2.7–3.1
Albumin (g/L)	43.9	40–55
AST (U/L)	26	15–40
ALT (U/L)	37.2	9–50
SHBG (nmol/L)	22	20.6–76.7
Growth hormone (ng/mL)	1.45	0.03–2.47
IGF-1 (ng/mL)	74.6	45–210
Cortisol at 10 AM (nmol/L)	139.6	101.2–535.7
Cortisol at 2 AM (nmol/L)	32.9	–
ACTH at 10 AM (pg/mL)	17.4	7.2–63.3
FSH (IU/L)	9.31	0.95–11.95
LH (IU/L)	4.75	0.57–12.07

Abbreviations: LDL-C: low-density lipoprotein cholesterol; AST: aspartate aminotransferase; ALT: alanine aminotransferase; SHBG: sex hormone-binding globulin; IGF-1: insulin-like growth factor-1; ACTH: adrenocorticotropic hormone; LH: luteinizing hormone; FSH: follicle-stimulating hormone.

**Figure 2:**
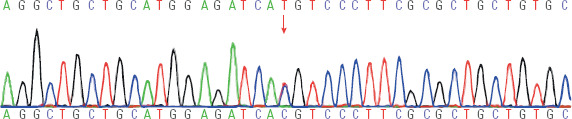
Sequencing analysis of the thyroid hormone receptor beta (THRB) gene in the proband. A heterozygous point mutation was identified in exon 9, where the nucleotide at position 938 was mutated from thymine to cytosine. This mutation resulted in the substitution of methionine with threonine at amino acid position 313. The arrow indicates the location of this point mutation.

In this study, whole–exome high–throughput sequencing (WES) was employed. The data were analyzed using the Verita Trekker variant detection system and the Enliven variant annotation and interpretation system, both of which were independently developed by Berry Genomics (Beijing, China). For suspected dynamic mutations identified by WES, a comprehensive analysis was conducted using polymerase chain reaction (PCR) and capillary electrophoresis. Based on the guidelines of the American College of Medical Genetics and Genomics (ACMG) and the application recommendations of the ClinGen Sequence Variant Interpretation (SVI) Expert Panel, as well as a review of public databases such as the Human Phenotype Ontology (HPO; https://hpo.jax.org/app/), Online Mendelian Inheritance in Man (OMIM; https://omim.org/), and Genetics Home Reference (https://medlineplus.gov/genetics/), the c.938T>C mutation in the *THRB* gene (Figure 2) was classified as a pathogenic mutation site.

## Discussion

First reported by Refetoff and cols. in 1967, RTHS is a rare endocrine disorder with an incidence of approximately 1 in 40,000 individuals. Here, we report a case of a 57–year–old male with RTHS. The patient presented with symptoms typical of hyperthyroidism, including palpitations and hand tremors. Thyroid function tests revealed elevated serum thyroid hormone levels, while TSH levels remained normal, consistent with the biochemical and clinical features commonly reported in RTHS ^(10,11)^. Genetic testing identified a novel mutation in the *THRB* gene. Specifically, a heterozygous point mutation was found at nucleotide position 938 in exon 9, where thymine was replaced by cytosine (c.938T>C). This mutation resulted in the substitution of methionine with threonine at amino acid position 313 (p.M313T). This mutation site is located in a key functional domain of the *THRB* gene, likely altering the receptor’s conformation and affecting its affinity for thyroid hormones. This, in turn, interferes with the receptor’s binding efficiency and transcriptional activity, ultimately leading to thyroid hormone resistance. This finding is consistent with previous studies showing that most RTHS cases are caused by *THRB* gene mutations and exhibit autosomal dominant inheritance ^(12,13)^. Functional studies of *THRB* gene mutations have provided strong evidence supporting this mechanism ^(14)^. To our knowledge, the specific mutation identified in this patient has not been previously reported.

Notably, RTHS is primarily driven by heterozygous pathogenic variants in the *THRB* gene, which account for over 80% of affected families. These mutations cluster in three critical hotspot regions (exons 7–10), including the segment encompassing codons 310–353, where our novel p.M313T variant is located ^(15)^. These regions are essential for ligand binding and signal transduction. Consequently, mutations like p.M313T are predicted to disrupt thyroid hormone binding affinity and subsequent transcriptional activity, leading to the clinical manifestation of hormone resistance. While *THRB* mutations represent the majority of cases, approximately 10% are attributed to other mechanisms, such as reduced number of thyroid hormone receptors or other unknown factors ^(16)^. The specific organ distribution of functional receptor isoforms (TRα1, TRβ1, and TRβ2) underlies the diverse clinical presentations observed in RTHS.

The clinical manifestations of RTHS are diverse and complex. Goiter is present in 66–95% of patients. Approximately 60% of patients have mood disorders, and 40–60% suffer from attention–deficit/hyperactivity disorder, presenting with behaviors such as inattentiveness, hyperactivity, and impulsivity. Additionally, 33–75% of patients exhibit tachycardia ^(17,18)^. Notably, goiter was absent in our patient.

Based on clinical manifestations, RTHS is classified into three main types: Generalized Resistance to Thyroid Hormone (GRTH), Pituitary Resistance to Thyroid Hormone (PRTH), and Peripheral Resistance to Thyroid Hormone (PRTH). Patients with GRTH usually have no obvious symptoms except for goiter. Patients with PRTH show mild to moderate manifestations of hyperthyroidism, such as hyperhidrosis, palpitations, and hand tremors, but may also present with symptoms of hypothyroidism. In our case, the patient presented with clinical symptoms of hyperthyroidism, including insomnia, tachycardia, and hand tremors. Therefore, the patient is considered to have PRTH. In addition to these symptoms, some patients may also experience growth retardation, decreased learning ability, hearing impairment, and abnormal bone development ^(19)^. The symptoms vary widely among different patients, and even among patients with the same mutation within the same family, the symptoms may not be identical. This clinical heterogeneity is likely due to the variable nature of the genetic mutations and the specific tissues affected.

Diagnosis of RTHS requires careful judgment by comprehensively considering clinical symptoms, physical signs, family history, and the results of other auxiliary examinations ^(20)^. Gene sequencing is the gold standard for diagnosis. In terms of differential diagnosis, RTHS has many similarities with other diseases that can lead to elevated thyroid hormone levels without TSH suppression and needs to be carefully distinguished. We retested the patient’s thyroid function in another hospital using different reagents. The results of the retest were consistent with those obtained in our hospital, thereby ruling out potential detection errors. Under such circumstances, it was necessary to differentiate whether our patient had a thyrotropin–secreting pituitary adenoma (TSHoma). Ninety percent of patients with TSHomas show a blunted response to thyrotropin–releasing hormone (TRH) stimulation. An MRI of the sella turcica often reveals a pituitary adenoma. There are no *THRB* gene mutations, and the serum level of sex hormone–binding globulin (SHBG) is usually high. In contrast, in most patients with RTHS, TSH shows a normal response to TRH stimulation, there is no pituitary adenoma, and approximately 80% of them have *THRB* gene mutations, with a normal serum SHBG level. In addition, it is also necessary to differentiate from diseases caused by mutations in the *THRA* gene. In patients with *THRA* mutations, the levels of thyroid hormones and TSH are close to normal, but growth and gastrointestinal function abnormalities are present, while in RTHS, the main feature is the reduced responsiveness of tissues to thyroid hormones. Enhanced MRI of the pituitary gland in our patient did not detect any pituitary adenoma. Additionally, serum SHBG level was within the normal range. Therefore, the diagnosis of TSHoma was excluded in our patient.

Having established the diagnosis of RTHS through differential diagnosis, the focus shifts to its clinical management. Regarding the treatment strategy, there is currently no radical cure for RTHS. The main treatment principles focus on effectively relieving patients’ symptoms and improving their quality of life. For patients with relatively mild symptoms, such as the one in this case, the use of beta blockers has achieved good results in relieving tachycardia. This is mainly because beta blockers can specifically block the excessive stimulation of thyroid hormones on cardiac β–receptors, thereby reducing the discomfort caused by a rapid heart rate and lowering the risk of cardiovascular events ^(21)^. Patients with RTHS must strictly avoid the use of antithyroid drugs (ATDs), primarily because the function of these drugs is to inhibit the synthesis of thyroid hormones, which is completely contrary to the compensatory mechanism of RTHS and would disrupt the body’s fragile compensatory balance. If ATD is used, they further reduce circulating thyroid hormone levels. In patients with reduced tissue responsiveness to thyroid hormone, additional lowering of hormone levels can further limit the availability of thyroid hormones to target tissues, potentially exacerbating symptoms of hypothyroidism, such as fatigue and lethargy. Simultaneously, the reduction in thyroid hormone levels stimulates the pituitary gland to secrete a large amount of TSH through negative feedback, potentially leading to pituitary TSH cell hyperplasia or even pituitary adenoma in the long term. It will also mask the typical laboratory features of RTHS, complicating the diagnosis and subsequent treatment direction. This has been fully demonstrated by Lai and cols. in relevant research ^(22)^. For patients with symptoms of hypothyroidism or growth retardation, thyroid hormone replacement therapy can be considered on the premise of closely monitoring thyroid function indicators. Furthermore, for patients with mood disorders or attention–deficit/hyperactivity disorder, psychological counseling and drug interventions can be implemented. However, during the treatment process, it is necessary to closely monitor the dynamic changes of thyroid function to prevent overtreatment and avoid other adverse reactions ^(23)^. Throughout the treatment, it is essential to closely monitor indicators, including thyroid hormone levels, TSH, and liver and kidney function, to adjust the treatment plan in a timely manner. At the same time, genetic screening of the patient’s relatives is helpful for early detection and treatment of potential patients.

Our study identified a novel heterozygous p.M313T mutation in the TRβ gene of a patient with generalized RTHβ. While the p.M313T variant itself has not been previously documented, its pathological relevance is strongly corroborated by a highly parallel finding in the literature. Notably, a study by Del Prete and cols. (2021) reported an identical methionine–to–threonine substitution — p.M310T — in a patient with a virtually identical phenotype of generalized RTHβ ^(24)^. This finding provides a crucial context for our discovery. The coexistence of pathogenic M→T mutations at adjacent codons 310 and 313 signifies that the M310–M313 segment constitutes a genuine and critical mutational hotspot within the ligand–binding domain of TRβ. The clustering of phenotypically identical missense mutations is a hallmark of functionally indispensable protein regions. The fact that both mutations involve the replacement of a bulky, hydrophobic methionine with a polar threonine suggests a common mechanistic basis for pathogenicity.

One limitation of the present study was the inability to perform genetic testing on the proband’s family members. This constraint has several important implications. First, it restricts our understanding of the inheritance pattern, as we cannot definitively distinguish whether the p.M313T variant is a de novo or an inherited mutation, which directly affects the assessment of its penetrance. Second, it introduces significant uncertainty into clinical genetic counseling, precluding accurate recurrence risk assessment for the patient’s first–degree relatives and future offspring. Finally, potential strategies for early detection and preventive management cannot be implemented, as we are unable to identify other potentially asymptomatic family members who may carry the pathogenic variant.

Several critical challenges in the RTHS field warrant further investigation. A primary focus should be on establishing large–scale, international patient registries. These multicenter databases are crucial for collecting robust, longitudinal data on this rare disorder. Furthermore, detailed genotype–phenotype correlation studies are needed to elucidate how specific mutations influence disease severity, clinical presentation, and long–term outcomes. On the clinical front, research must prioritize the development of standardized strategies for early detection of RTHS, including genetic screening protocols for at–risk relatives and refined diagnostic algorithms for patients with unexplained thyroid function test abnormalities. Future research combining these clinical findings with foundational studies on molecular pathogenesis will be key to developing personalized management plans and improving overall patient prognosis and quality of life.

## CONCLUSION

We report a case of a 57–year–old Chinese male with RTHS. A novel heterozygous point mutation — c.938T>C: p.M313T — in the *THRB* gene was discovered. This finding further enriches the clinical case data of RTHS in the Chinese population and is of great significance for improving clinicians’ awareness and diagnostic capabilities regarding this disease.

## Data Availability

datasets related to this article will be available upon request to the corresponding author.
